# Chondrogenesis of human amniotic fluid stem cells in Chitosan-Xanthan scaffold for cartilage tissue engineering

**DOI:** 10.1038/s41598-021-82341-x

**Published:** 2021-02-04

**Authors:** Carolina C. Zuliani, Ingrid I. Damas, Kleber C. Andrade, Cecília B. Westin, Ângela M. Moraes, Ibsen Bellini Coimbra

**Affiliations:** 1 Rheumatology Unit, Department of Clinical Medicine, School of Medical Sciences, State University of Campinas (UNICAMP), 126 Tessália Vieira de Camargo Street, Campinas, SP CEP 13083-887 Brazil; 2 Department of Gynecology and Obstetrics, School of Medicine, State University of Campinas (UNICAMP), 101 Alexander Fleming Street, Campinas, SP CEP 13083-891 Brazil; 3 Department of Materials Engineering and Bioprocesses, School of Chemical Engineering, State University of Campinas (UNICAMP), 500 Albert Einstein Avenue, Campinas, SP CEP 13083-852 Brazil

**Keywords:** Biotechnology, Stem cells, Rheumatology

## Abstract

Articular chondral lesions, caused either by trauma or chronic cartilage diseases such as osteoarthritis, present very low ability to self-regenerate. Thus, their current management is basically symptomatic, progressing very often to invasive procedures or even arthroplasties. The use of amniotic fluid stem cells (AFSCs), due to their multipotentiality and plasticity, associated with scaffolds, is a promising alternative for the reconstruction of articular cartilage. Therefore, this study aimed to investigate the chondrogenic potential of AFSCs in a micromass system (high-density cell culture) under insulin-like growth factor 1 (IGF-1) stimuli, as well as to look at their potential to differentiate directly when cultured in a porous chitosan-xanthan (CX) scaffold. The experiments were performed with a CD117 positive cell population, with expression of markers (CD117, SSEA-4, Oct-4 and NANOG), selected from AFSCs, after immunomagnetic separation. The cells were cultured in both a micromass system and directly in the scaffold, in the presence of IGF-1. Differentiation to chondrocytes was confirmed by histology and by using immunohistochemistry. The construct cell-scaffold was also analyzed by scanning electron microscopy (SEM). The results demonstrated the chondrogenic potential of AFSCs cultivated directly in CX scaffolds and also in the micromass system. Such findings support and stimulate future studies using these constructs in osteoarthritic animal models.

## Introduction

Injuries to articular cartilage can be caused mainly by traumas, such as those occurring in athletes or by diseases that can affect this tissue, such as osteoarthritis (OA)^1,2^. In people over 65 years old affected by OA, tissue degeneration is, at least in part, responsible for clinical manifestations such as pain and functional incapacity, leading to high costs for health systems^3^. Chondral lesions have been diagnosed earlier and at a higher frequency. However, the available treatments are palliative, focused on the relief of symptoms and without robust evidence of modification in disease progression^4^.

Several sources of stem cells have been studied for the regeneration of different tissues. In the case of hyaline cartilage, cells from adipose tissue^5–7^, bone marrow^7,8^, umbilical cord blood^9,10^, periosteum^11,12^, dental pulp^13^, placenta^14^, amniotic fluid^7,15^ and embryos^16^ have already been investigated. Among these many possibilities, the potential use of amniotic fluid stem cells (AFSCs) has attracted attention due to their low immunogenicity and tumorigenic characteristics when implanted in vivo and due to the absence of ethical problems, since these cells can be collected during routine amniocentesis procedures. In addition, these cells are isolated by adherence to culture flasks and are easily expanded^17^. AFSCs can differentiate into cells of the three germ layers (endoderm, mesoderm, and ectoderm) and have recently been shown to be able to become pluripotent, the so-called induced pluripotent stem cells (iPSCs)^18^. AF has great variability of cell types with characteristics of intermediate phenotype between mesenchymal stem cells (MSCs)^19^ and embryonic stem cells (ESCs). The amniotic fluid is composed of water, different types of chemicals and heterogeneous populations of cell types. The cells present in AF during the second trimester of pregnancy are mainly of fetal origin and are classified into and subpopulations according to their morphology^17,20^. Among those of fetal origin, a subpopulation of undifferentiated cells can be characterized by the expression of the surface marker c-kit (CD117) or type III tyrosine kinase receptor for stem cell factor^21^. In addition, this type of cell shows expression of markers such as Octamer transcription factor-4 (Oct-4)^22^ and Stage-specific embryonic antigen-4 (SSEA-4)^20,21^. Furthermore, these cells have high proliferation rates and maintain their undifferentiated phenotype even after many passages^23,24^.

The use of stem cells allied to biocompatible and biodegradable three-dimensional scaffolds to heal injured regions seems to be an interesting solution^25,26^. Numerous materials have been investigated as potential scaffolds. These include both synthetic materials, such as polystyrene, poly-l-lactic acid (PLLA)^27^, polyglycolic acid (PGA)^28^ polylactic-co-glycolic acid (PLGA), and polyethersulfone (PES)^29^—or natural materials, mainly those produced with natural polysaccharides such as agarose, alginate, and chitosan, which present ideal properties for stem cell (SC) chondrogenesis^30^.

Chitosan is a polymeric biomaterial derived from the deacetylation of chitin, which, in turn, can be obtained from crustacean shells. Above its isoelectric point, chitosan becomes positively charged, which makes it capable of forming polyelectrolyte complexes with negatively charged polymers such as xanthan gum, a polysaccharide naturally synthesized by the bacterium *Xanthomonas campestris* and widely used in the pharmaceutical and food industries^31^. This study aimed to demonstrate chondrogenic differentiation of human amniotic fluid stem cells induced by insulin-like growth factor 1 (IGF-1) after high-density culture in a chitosan-xanthan gum (CX) support, aiming at the production bio-dressing for the treatment of small lesions in articular cartilage^32^.

## Results

### Characterization of AFSCs

**Figure 1:**
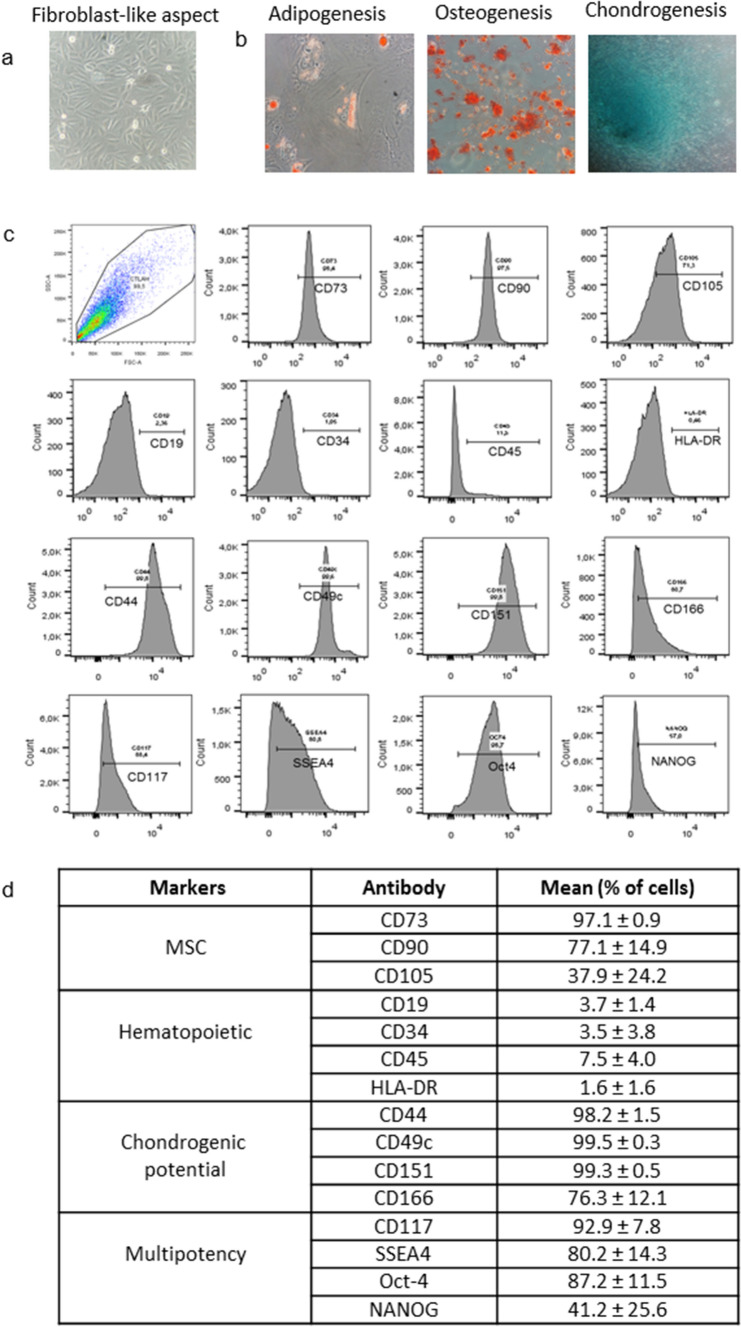
Characterization of human AFSCs: (**a**) cells showing adherent growth, exhibiting fibroblast-like aspect (400 ×); (**b**) differentiation potential in mesenchymal lineage after culture in specific medium (400 ×): adipogenic, oil Red O staining indicating lipidic vesicles; osteogenic, alizarin red staining indicating calcium matrix formation and chondrogenic, Alcian blue staining evidencing glycosaminoglycan presence; (**c**) immunophenotype analysis of AFSCs by flow cytometry; (**d**) percentage of cells showing specific markers of MSC, hematopoietic, multipotency, and chondrogenic potential in different samples.

The cells tested fulfilled all three criteria established by the consensus of the International Society for Cellular Therapy for characterization of MSCs^33^ (Fig. 1).

Microscopic analysis showed adhesion of the cells to the surface of the polystyrene culture flasks and typical fibroblast-like aspect (Fig. 1a). Under specific stimuli, they were able to differentiate in vitro into cells of the three major mesenchymal lines. Cultures with adipogenic stimulation exhibited fat vacuoles inside oil red stained cells. Cells with osteogenic stimulation showed abundant calcium matrix formation observed in red by alizarin red staining, and those stimulated with chondrogenic medium presented formation of a rich GAGs matrix stained by Alcian blue (Fig. 1b).

As observed in Fig. 1c and summarized in 1 day, the immunophenotypic characterization of AFSCs, after magnetic separation, showed positivity for CD90, CD105, and CD73 mesenchymal stem cell markers and negativity for hematopoietic lineage markers CD19, CD34, CD45, and HLA-DR. The cells also showed positivity for CD44, CD49c, CD151, and CD166 cell condensation markers as well as for Oct-4, NANOG, SSEA-4, and CD117 markers.

### Chondrogenesis in micromass culture

**Figure 2:**
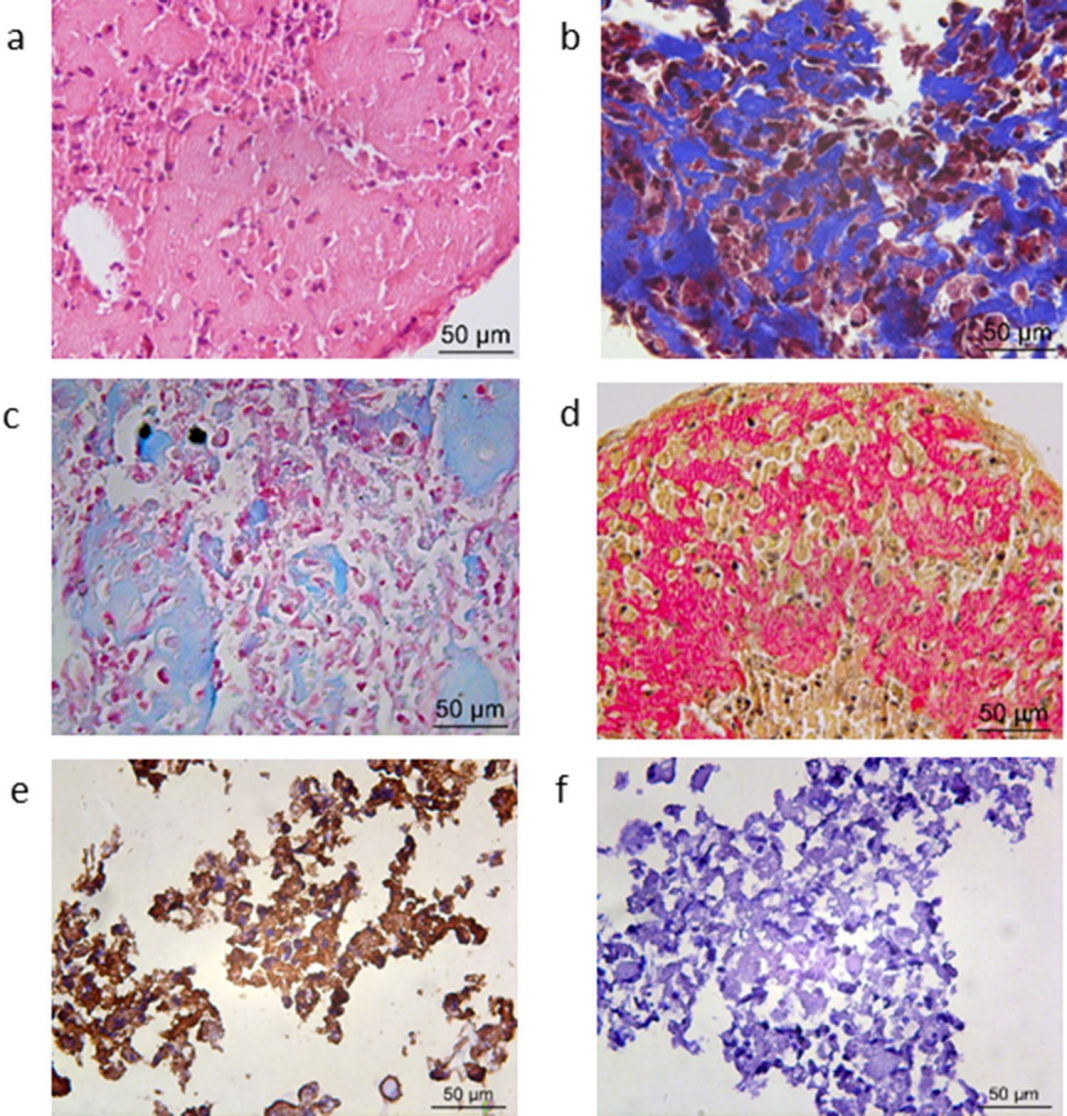
Micromass results stimulated with IGF-1 for 21 days by chondrogenic differentiation: Histological analysis (400 ×): H&E stain (neoformed matrix appears in pink) (**a**), Masson’s trichrome (collagens appear in blue) (**b**), Alcian Blue stain (glycosaminoglycans appear in light blue) (**c**), and Picrosirius Red stain (collagens fibers appears in red) (**d**); positive immunohistochemical staining for specific Type II collagen (**e**) and negative control (**f**).

After 21 days of culture, macroscopic whitish and shiny structures were observed. The histological analysis in sections stained by H&E (Fig. 2a), Masson's trichrome (Fig. 2b), Alcian blue (Fig. 2c), and Picrosirius red (Fig. 2d) revealed abundant matrix formation, suggestive of collagen and glycosaminoglycans (GAGs), the main components of the extracellular matrix of cartilage. The presence of type II collagen was confirmed by immunohistochemistry in structure cuts (Fig. 2e) using polyclonal anti-collagen II antibody.

### Chondrogenic differentiation of CD117^+^ AFSCs directly in the CX scaffold

**Figure 3:**
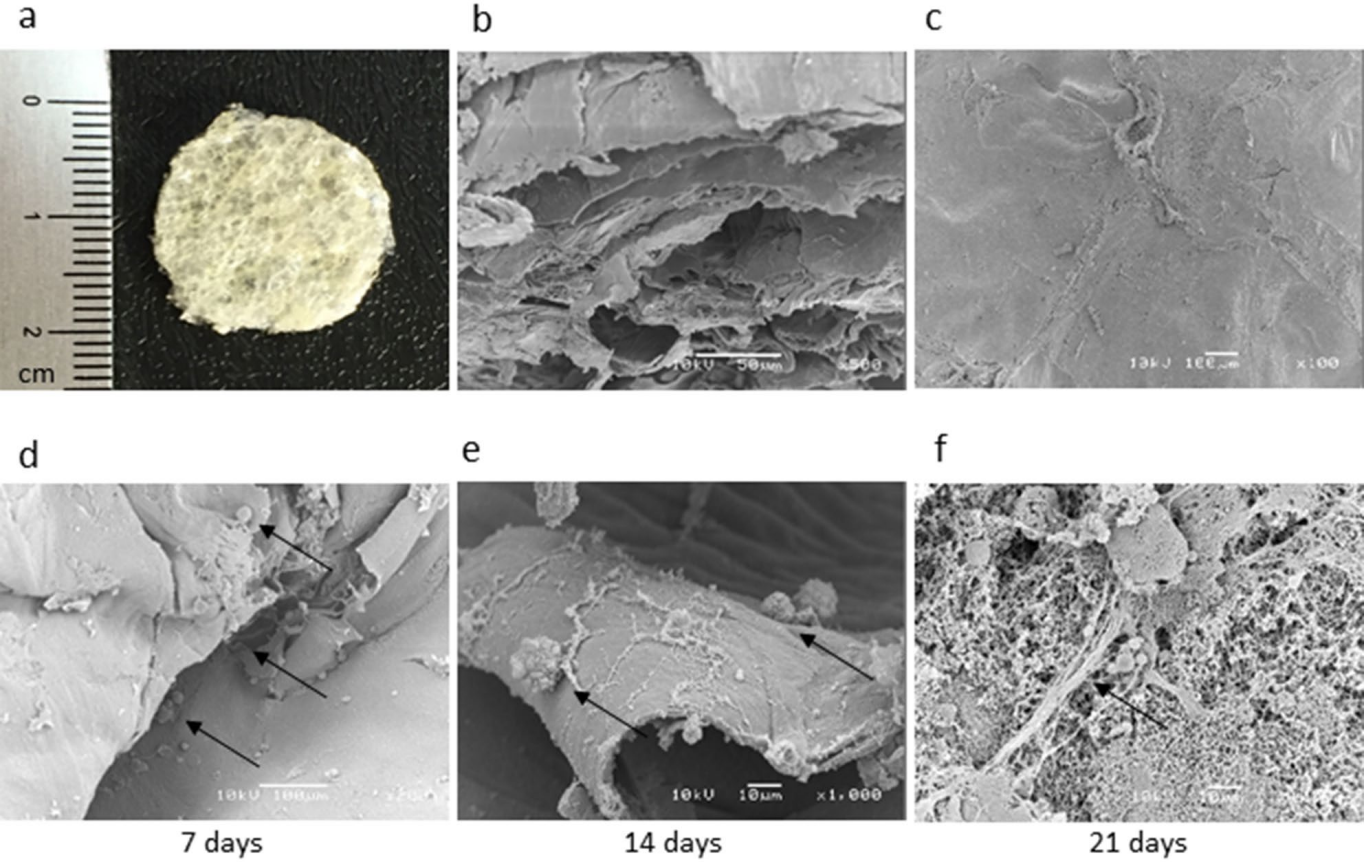
Macroscopic aspect of the CX scaffold (**a**) SEM micrographs of CX: section porous structure (**b**); surface and morphology (**c**). Aspect of CD117^+^ AFSCs cultured in CX analyzed in different periods. The black arrows indicate the cells adhered to the scaffold with 7 days (**d**), 14 days (**e**), and 21 days of culture, exhibiting many adhered cells and evident matrix network production (**f**).

**Figure 4:**
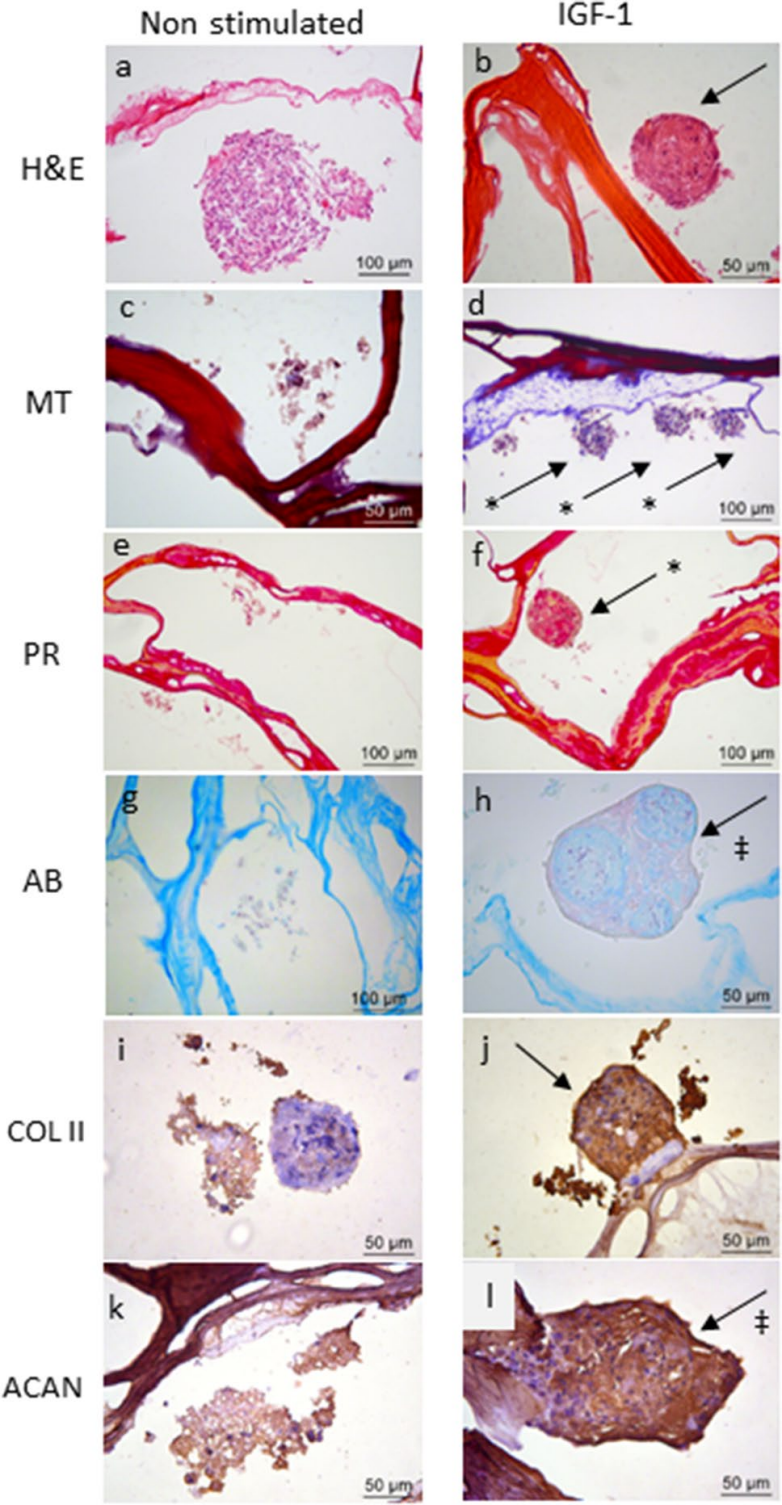
Chondrogenic differentiation of CD117^+^ AFSCs seeded into CX scaffold for 21 days with and without IGF-1 stimulation. In the group stimulated by IGF-1 (**b**,**d**,**f**,**h**,**j**,**l**) more compact agglomeration of the cells is observed,with a higher amount of extracellular material compared to that noticed in the non-stimulated control group (**a**,**c**,**e**,**g**,**i**,**k**). H&E staining (**a**,**b**); collagen (indicated with *) stained in blue by Masson´s trichrome (MT) (**c**,**d**); collagen fibers shown in red by Picrosirius red (PR) (**e**,**f**); glycosaminoglycans (‡) stained light blue using Alcian blue (AB) (**g**,**h**). Labeling with anti-collagen type II antibody (COL II) (**i**,**j**) and anti-aggrecan antibody (ACAN) (**k**,**l**) in the stimulated group compared to the control group.

The scaffolds used proved to be flexible, opaque, corrugated, and porous (Fig. 3a–c), with thickness between 887 and 969 μm. Cytotoxicity assays by MTT technique demonstrated that daily replacements of the culture media were required for 7 days prior to cell inoculation to attain complete hydration and pH stabilization of the biomaterial. Representative images of the scaffold are shown in Fig. 3 along with typical scanning electron microscopy results of the cells 7, 14, and 21 days after inoculation (Fig. 3d–f). Structures similar to those seen in the histology and immunohistochemical slides were observed in Fig. 4, indicating adherence of the cells to the tested material, intense cell growth, and evident extracellular matrix network production.

After 21 days, the CX constructs cell-scaffold, stimulated or not with IGF-1, were analyzed histologically, and the representative results are shown in Fig. 4 for both groups. H&E staining (Fig. 4a,b) indicated that the cells remained viable inside the material, with intact cells with purplish nuclei and reddish cytoplasm. Based on specific staining for cartilaginous tissue, the group of IGF-1 stimulated cells presented differentiation with collagen production, confirmed by blue staining among the cells in the sections stained by MT (Fig. 4d), and a network of reddish when stained by Picrosirius red (Fig. 4f). The production of GAGs, stained light blue among the cells, was also observed in sections stained by Alcian blue (Fig. 4h). In cells without stimulation (control group), a small and discrete production of collagen in the MT slides was observed (Fig. 4c), probably due to the micromass-like grouping effect.

In sections incubated with anti-collagen II and anti-aggrecan antibodies, positive labeling was observed in both experimental groups (Fig. 4i–l), without and with IGF-1 stimulus. Nevertheless, the latter appeared to be more intense than that observed in the non-stimulated group. The negative control did not show any marking (results not shown).

## Discussion

This study demonstrates that it is possible to differentiate stem cells from human amniotic fluid into chondrocytes when seeded directly in an efficient and low-cost CX scaffold produced by the combination of two renewable biopolymers, chitosan and xanthan gum, as compared to cells cultured according to the micromass approach. It is a promising biomaterial for the treatment and repair of small articular cartilage lesions associated with trauma or diseases such as osteoarthritis^1^.

As noted herein, AFSCs show high cell proliferation in culture without loss of phenotype, as well as multipotency or partial pluripotency characteristics confirmed by the presence of typical ESC markers (Oct4, SSEA4, c-kit, or CD117)^20^. In addition, these cells show the ability to differentiate into the major mesodermal lines and, when compared to other sources of stem cells, have lower immunogenicity and are not tumorigenic^19,20,34^. It is also noteworthy that they can be obtained in routine examinations for prenatal genetic diagnosis from the backup samples without increasing the low risk of the procedure itself, for which ethical concerns are not a significant restriction compared to the collection of embryonic tissue^20^. Such characteristics indicate that their clinical use could be important and strategic in the treatment of articular cartilage regeneration. In a recent review^17^, several studies showed evidence of the potential use of AFSCs in clinical applications related to cell therapy in different areas such as hematogenous, gastrointestinal, cardiovascular, nervous, respiratory, urinary, and musculoskeletal. However, little research has been done on AFSCs chondrogenic properties applied to tissue engineering, which makes further studies in this area particularly important in view of the low potential for spontaneous healing of cartilaginous tissue.

This study presented a great variability of cell lines in the amniotic fluid, which made it necessary to perform two cell selection steps to obtain a more homogeneous population. Initially, only adherent cells were analyzed, and subsequently surface antigen positive cells separated using a CD117 (c-Kit) magnetic immunoassay were investigated. As found in other studies^21,24^, our results also confirm that CD117^+^ AFSCs have all the minimum criteria for classification as human MSCs^33^. However, unlike expected by the proposed consensus, the presence of CD34 and CD45 in low levels of expression was detected, which was also reported by Ditadi et al.^35^. Likewise, in a recent study^23^ based on the analysis of 165 AF samples, both CD34 and CD45 were expressed in cells and the amount of these markers increased with the number of passages, while CD90 decreased. In our study, the cytometric analyses were performed after four passages, and the quantities found for these markers were similar and compatible with those found in that study. Perhaps these findings may be justified by the fact that the fetal stem cells found in the amniotic fluid, notably in the early stages of gestation, largely resemble embryonic cells and, as recently suggested by Loukogeorgakis and De Coppi^19^, those cells would present an intermediate cellular phenotype between ESCs and adult MSCs.

Flow cytometry analysis also showed high levels of cell condensation markers expression: CD49c (α3 integrin), the major CD44 (hyaluronan receptor), CD151 (tetraspanins), and CD166, all indicative of CD147^+^ AFSCs chondrogenic potential. These findings were important for the purpose of this study. Subpopulations of chondrocytes express high levels of membrane markers and genes involved in cell–matrix interactions essential to the onset of chondrogenesis, with formation of cartilaginous tissue with higher levels of collagen type II and GAGs^36^. Initially, CD117^+^ AFSCs were cultured for 21 days in chondrogenic medium in the presence of IGF-1 at 10 ng/ml in a high-density micromass culture. Previous studies from our group have shown the efficiency of this system in the induction of chondrogenesis from umbilical cord MSCs and AFSCs under the influence of TGFβ3^9,11,37^. In this study, after 21 days of culture, it was possible to show the formation of whitish pellets with hardened consistency, suggesting, at least macroscopically, the production of a tissue very similar to hyaline cartilage. When analyzed histologically, cellular aggregates permeated by abundant extracellular material suggestive of cell matrix production were observed (Fig. 2). Section treatment with specific cartilage staining evidenced the formation of collagen fibers stained in blue by Masson's trichrome (Fig. 2b) and varying in intensity from pale yellow to intense red according to Picrosirius staining (Fig. 2d). Likewise, the formation of glycosaminoglycans in light blue was evidenced by Alcian blue staining (Fig. 2c), which, as well as collagen, is a fundamental component of cartilage. In addition to demonstrating matrix formation, it was important to prove that the collagen fibers produced were type II collagen, the most specific type of hyaline cartilage, by immunohistochemistry analysis (Fig. 2e), thus proving in an unprecedented way the chondrogenic differentiation of these cells in this system under IGF-1 stimulation, similarly to that observed previously by our group under the stimulation of TGF-β3^15^.

In a second step, the CD117^+^ AFSCs were injected directly into the porous scaffold of chitosan-xanthan. The choice of this scaffold was based on the characteristics of chitosan as a biopolymer widely used in tissue engineering due to its high availability, biocompatibility, and biodegradability. The experience of our group with chitosan-xanthan scaffolds demonstrated their effectiveness when combined with mesenchymal stem cells in the treatment of cutaneous lesions^38^. The physical–chemical characterization of the scaffold used was previously described by our group and is reported in detail elsewhere^13^. The experiments performed in the present studies pointed out initially to low scaffold cytotoxicity for CD117^+^ AFSCs. However, consecutive washings with culture medium were able to circumvent the problem associated to the initial low matrix pH.

In the histological analysis of the pH stabilized matrix and cells construct, viable cells well distributed throughout the internal pores of the scaffold, as well as on its surface, were observed, showing extensive formation of collagen-glycosaminoglycan extracellular matrix. These findings were even more evident with positive immunoblotting by anti-Collagen type II and anti-Aggrecan antibodies. Images obtained from SEM confirmed these results of an abundant number of cells with rich extracellular matrix network formation well distributed both on the surface of and inside the scaffolds. The findings were similar to those of a previously published study in which the same scaffold was used, but with stem cells collected from human dental pulp stimulated for chondrogenic differentiation with kartogenin^13^. Similarly, some studies have been successful with the use of chitosan and silk fibroin scaffolds to culture adult bovine chondrocytes^39^ or mesenchymal stem cells from mouse bone marrow^40^ as a promising approach to tissue engineering to repair defects of cartilage and to study the formation of cartilaginous tissue in vitro. Recent studies using different scaffolds based on PES, either alone or with polyaniline (PANI) blend, showed chondrogenesis from human adipose tissue cells^41^, bone marrow mesenchymal stem cells (BMSCs)^42^, as well as induced iPSC chondrogenesis isolated or under TGF-β3 stimuli^29,43^. Kolambkar et al.^44^ in 2007 investigated the differentiation of hAFSC in chondrocytes using a total AFSC cell population in pellets and in alginate hydrogel, differently from ours, with CD117 + cells selected and differentiation in micromass and directly in a chitosan-xanthan scaffold.

Finally, this study made it possible to show for the first time the efficacy of human AFSCs as an important source for the induction of chitosan-based scaffold chondrogenesis. Limitations of this study include lack of a quantitative method to determine the expression of cartilage-specific genes and hypertrophy markers, such as type X collagen, to better evaluate cell differentiation. Also, functional studies of chondral lesions in animal models to confirm the usefulness of this cellular dressing for future clinical use of the construct in humans are needed. Considering the positive results obtained, we believed that, in a next step, it would be important to standardize a quantitative method and to evaluate the scaffold-cell performance in a chondral lesion animal model.

## Methods

All experimental protocols, including informed consent, were approved by the Ethical Committee of the University of Campinas, São Paulo, Brazil (approval CAAE: 31984414.6.0000.5404). All the patients provided written informed consent for the amniocentesis and the use of samples and data for research purposes. All experiments were performed in accordance with relevant guidelines and regulations, including the Declaration of Helsinki—Ethical Principles for Medical Research Involving Human Subjects.

### Isolation, expansion, and selection of CD117^+^ human amniotic fluid stem cells

Amniotic fluid samples were obtained from routine amniocentesis performed during the 2nd trimester of gestation from 43 women at the Center for Integrated Health Care for Women (CAISM), University of Campinas (Campinas, SP, Brazil). Aliquots of 10 mL of human amniotic fluid (AF) were obtained from each woman and, after karyotype analysis, only those not showing alterations were used in the study, in a total of 22. Samples from four women were used in experiments standardization, eight in micromass differentiation, and ten in scaffold differentiation. The harvested material was centrifuged at 300G for 10 min and the precipitate was then transferred to 25 cm^2^ T-Flasks with MEM α (Minimum Essential Medium α) (Gibco) for cell expansion supplemented with 20% fetal bovine serum (Gibco) and 1% penicillin/streptomycin solution (Gibco). The cells were incubated at 37 °C with 5% CO_2_. After 6 days, non-adherent cells were discarded and the culture medium was exchanged. Upon reaching 75% confluency, the cells were detached using 0.25% trypsin/EDTA solution (Gibco) for 5 min at 37 °C and transferred to larger T-Flasks. After the cells reached 75% confluency, they were labeled with specific antibody and a magnetic immuno-separation procedure of CD117 (c-kit) positive cells^21^ was performed, according to the manufacturer's protocol (Human CD117 MicroBead kit-MACS-Miltenyi Biotec). The CD117^+^ AFSCs were cultured up to the fourth passage for characterizations and chondrogenesis experiments using the micromass culture system and the chitosan-xanthan gum scaffold.

### Characterization of CD117^+^ AFSCs

Samples from four women were used in this step. The cells were evaluated according to the criteria for characterization of MSCs^33^. Regarding adhesion to polystyrene and formation of fibroblast-like forming colonies, immunophenotypic^15^ characterization was done by flow cytometry by labeling the cells with the following antibodies: CD73-PECy7, CD90-FITC, CD105- FITC, CD19-PE Cy7, CD34-APC Cy7, CD45-APC, HLA-DR-PERCP Cy5.5, CD44-PERCP Cy5.5, CD49c-PE, CD151-APC, CD166-PE, CD117–APC, SSEA4-FITC, Oct4-PE (Biolegend, San Diego, CA), and NANOG-PERCP Cy5.5 (BD Pharmingen). The analysis was done in BD FACSCanto or BD FACSVerse and the results were analyzed using BD FACSDiva.

Analysis of differentiation into the three main mesenchymal lines was performed using adipogenesis, chondrogenesis, and osteogenesis StemPro differentiation kits (Gibco), according to the manufacturer's protocol. 0.5% oil Red O (Sigma-Aldrich), Alcian blue (Sigma-Aldrich), and Alizarin red (Sigma-Aldrich) were used respectively to observe adipogenesis, chondrogenesis, and osteogenesis.

### Differentiation of CD117^+^ AFSCs in micromass culture

Eight AFSC samples from different women were used in this step. The cells were differentiated in a high-density micromass culture^45^ upon stimulation with IGF-1. After expansion, the cells were centrifuged at 300*g* for 10 min, diluted to 15–20 × 10^6^ cells/ml, and then approximately 3 × 10^5^ cells were placed in a 96-well V-shaped bottom plate (Corning) and incubated at 37 °C and 5% CO_2_ for 2 h to allow them to adhere to the wells. Next, 0.2 mL of chondrogenic medium composed of Dulbecco's modified Eagle's medium glucose (Gibco), supplemented with 50 μg/mL ascorbic acid (Sigma-Aldrich), 40 μg/mL proline (Sigma-Aldrich), 1% insulin-transferrin-selenium (ITS + 1) (Sigma-Aldrich), 0.1 μmol/L dexamethasone (Sigma-Aldrich), and 10 ng/mL IGF-1 growth factor (R&D Systems), was gently added to the wells. The cells were cultured during 21 days under the same conditions with exchanges of culture medium every 3 or 4 days.

### Differentiation of CD117^+^ AFSCs directly in chitosan-xanthan scaffold

Cells from 10 different women were used in this step. The CX scaffold was prepared using chitosan and xanthan at a ratio of 1:1, as described by Bellini et al.^38^ and adapted by Westin et al.^13^. Indirect toxicity of CX scaffolds to AFSCs was assessed by the colorimetric MTT (3-(4,5-dimethylthiazol-2-yl)-2,5-diphenyltetrazolium bromide), according to methodology previously published by the authors^13^.

Circular scaffolds of 1.5-cm diameter were placed in 24-well plates and sterilized with ethylene oxide (EO) by exposure to Oxyfume-30 (30% EO and 70% carbon dioxide) for 8 h at 40 °C and relative humidity of 30–80% at Acecil Central de Esterilização Comércio e Indústria (Campinas, SP, Brazil).

The sterilized scaffolds were hydrated in high-glucose DMEM culture medium and incubated at 37 °C and 5% CO_2_. The medium was replaced every 24 h for 7 days for pH neutralization. The cells from 10 samples in the fourth passage were injected into the scaffolds at a concentration of 2 × 10^6^ cells diluted in 0.5 mL of chondrogenic medium containing IGF-1 and cultured for 21 days with exchanges of culture medium every 3 or 4 days.

### Confirmation of differentiation

After 21 days, cells collected both from the micromass and CX scaffolds were analyzed histologically by optical microscopy (Leica DM2500) with the aid of Leica Application Suite (LAS) software (version 4.6.2) after staining with Hematoxylin and eosin (H&E), Masson's trichrome (MT), and Picrosirius red (PR) to determine the presence of collagen, and with Alcian blue (AB) to detect glycosaminoglycans (GAG), according to previously described specific protocols^46^.

Immunohistochemistry tests were performed in different samples. Sections were immersed in 1% Trilogy solution (Cell Marque) and placed in a steamer for 15 min for deparaffinization, rehydration, and recovery of the antigens. Endogenous peroxidase was blocked with H_2_O_2_, and each section was incubated with rabbit anti-collagen II polyclonal antibody (Bioss, catalog bs-0709R) and rabbit anti-aggrecan polyclonal antibody (Bioss, Catalog bs-11655R) at a 1:100 dilution ratio, overnight at 4 °C. Subsequently, the sections were treated with the HiDef Detection HRP Polymer System (Cell Marque), according to the manufacturer´s instructions. Color development was carried out with DAB solution and the material was counterstained with Harris hematoxylin, dehydrated, and mounted for microscopic analysis. A negative control slide was prepared without using antibodies.

Scaffold morphology was analyzed before and after the inoculation of cells at different culture times by scanning electron microscopy (SEM), according to a previously established protocol^13,47^.
